# Prediction of itraconazole minimum inhibitory concentration for *Fonsecaea pedrosoi* using Fourier Transform Infrared Spectroscopy (FTIR) and chemometrics

**DOI:** 10.1371/journal.pone.0243231

**Published:** 2020-12-02

**Authors:** Alessandra Koehler, Valeriano Antonio Corbellini, Daiane Heidrich, Maria Lúcia Scroferneker

**Affiliations:** 1 Postgraduate Program in Medicine: Medical Sciences, Universidade Federal do Rio Grande do Sul, Porto Alegre, Rio Grande do Sul, Brazil; 2 Department of Sciences, Humanities and Education, Postgraduate Program in Health Promotion, Postgraduate Program in Environmental Technology, Universidade de Santa Cruz do Sul, Santa Cruz do Sul, Rio Grande do Sul, Brazil; 3 Department of Microbiology, Immunology and Parasitology, ICBS, Universidade Federal do Rio Grande do Sul, Porto Alegre, Rio Grande do Sul, Brazil; Aligarh Muslim University, INDIA

## Abstract

*Fonsecaea pedrosoi* is one of the main agents of chromoblastomycosis, a chronic subcutaneous mycosis. Itraconazole (ITC) is the most used antifungal in its treatment, however, *in vitro* antifungal susceptibility tests are important to define the best therapy. These tests are standardized by the Clinical and Laboratory Standards Institute (CLSI), but these protocols have limitations such as the high complexity, cost and time to conduct. An alternative to *in vitro* susceptibility test, which overcomes these limitations, is FTIR. This study determined the minimum inhibitory concentration (MIC) of itraconazole for *F*. *pedrosoi*, using FTIR and chemometrics. The susceptibility to ITC of 36 strains of *F*. *pedrosoi* was determined according to CLSI and with the addition of tricyclazole (TCZ), to inhibit 1,8-dihydroxynaphthalene (DHN)-melanin biosynthesis. Strains were grown in Sabouraud agar and prepared for Attenuated Total Reflection (ATR)/FTIR. Partial least squares (PLS) regression was performed using leave-one-out cross-validation (by steps of quintuplicates), then tested on an external validation set. A coefficient of determination (R²) higher than 0.99 was obtained for both the MIC-ITC and MIC-ITC+TCZ ATR/PLS models, confirming a high correlation of the reference values with the ones predicted using the FTIR spectra. This is the first study to propose the use of FTIR and chemometric analyses according to the M38-A2 CLSI protocol to predict ITC MICs of *F*. *pedrosoi*. Considering the limitations of the conventional methods to test *in vitro* susceptibility, this is a promising methodology to be used for other microorganisms and drugs.

## Introduction

*Fonsecaea pedrosoi* is a dematiaceous filamentous fungus found in soil, plants and decomposing wood. This fungus is the main agent of chromoblastomycosis (CBM), a chronic, disabling and recalcitrant subcutaneous mycosis that occurs mainly in tropical regions [1]. The main virulence and protective factor of CBM agents is the 1,8-dihydroxynaphthalene (DHN)-melanin [2, 3]. The synthesis of DHN-melanin can be inhibited *in vitro* by the agrochemical tricyclazole ([1,2,4]triazolo[3,4-b][1,3]benzothiazole) [1].

Treatment is based on antifungal therapy, which can be combined with physical and surgical methods. Severe cases are difficult to treat due to the recalcitrant nature of CBM, and lesions caused by *F*. *pedrosoi* generally resist treatment [4]. The most used antifungal is itraconazole, but no randomized clinical trials have been conducted to determine the best therapy [1]. *In vitro* antifungal susceptibility testing may assist in that choice. Reference methods such as those recommended by the CLSI are highly complex, costly and require long periods to conduct [5]. Furthermore, they are still not standardized for CBM agents.

Considering conventional methods limitations, new methods to test *in vitro* susceptibility have been developed and Fourier Transform Infrared Spectroscopy (FTIR) has great potential. This technique generates containing the sample fingerprint, and has extensive applications in the biomedical field [6]. Its main advantages are the use of a very small amount of sample, preservation of the sample during analysis and minimal need for preparation, small or no waste generation, speed of analysis, accuracy, reproducibility and storage and data manipulation directly in the computer software [7].

The few published studies that have used FTIR for susceptibility analyses have reported promising results [8–11]. There are still no studies of this nature with filamentous fungi. In the case of CBM agents, for which susceptibility tests are carried out following the M38 CLSI reference method [12, 13], the use of FTIR may have numerous advantages, especially regarding cost, labor and waste generation. The aim of this study was to develop a prediction model for itraconazole MICs of *F*. *pedrosoi* using FTIR and chemometric analysis, that showed promising results.

## Materials and methods

### Microorganisms

Thirty-six clinical *F*. *pedrosoi* strains from the fungi collection of Laboratory of Pathogenic Fungi, Department of Microbiology, ICBS, UFRGS were studied. All strains were previously identified by sequencing ITS1-5,8S-ITS2 DNA region and deposited in GenBank® database (Table 1).

**Table 1:** Isolates used in the study, GenBank® accession numbers and MIC values (unpublished results).

GenBank® accession number	MIC ITC (mg/L)	MIC ITC+TCZ (mg/L)
MH382049	2	0.5
MH382054	0.5	0.5*
MH382034	0.125*	0.5
MH382053	1	0.5*
MH444810	0.25	0.5
MH382089	0.5*	0.25*
MH382042	0.5	0.25
MH382043	32*	1*
MH382046	32	1
MH382052	0.5*	0.25*
MH382083	0.5	0.125
MH382029	0.5*	0.5*
MH382030	0.5	0.5
MH382081	2*	0.5*
MH382032	0.5*	0.25
MH382033	32*	1*
MH382035	1*	0.5
MH382036	1	0.06*
MH382028	2	1
MH382047	0.5	0.5*
MH382080	1*	0.5
MH382045	0.5*	1*
MH382051	2*	1
MH368488	0.5	0.5*
MH382037	1	0.5
MH382038	1*	0.5*
MH382039	2	0.5
MH382040	0.5*	0.5*
MH382086	0.5	0.5
MH382041	0.5*	0.5*
MH382085	1	0.5
MH382050	1*	0.5*
MH382044	32	0.5
MH444807	1	1
MH382048	1*	2*
MH382087	0.25*	0.25*

MIC = minimum inhibitory concentration; ITC = itraconazole; TCZ = tricyclazole;

* = samples for calibration set.

*F*. *pedrosoi* itraconazole MICs were determined previously (unpublished results) using the microdilution technique according to CLSI protocol M38-A2, with a final antifungal concentration varying between 0.0625 and 32 mg/L. For each isolate, ITC MICs were also determined with the addition of 16 μg/mL of tricyclazole (Table 1).

#### Sample preparation for FTIR analysis

The 36 strains tested for itraconazole susceptibility with CLSI protocol were grown in potato dextrose agar (PDA) (Merck KGaA, Darmstadt, Germany) at 30°C for 14 days. After this period, conidial suspensions were prepared scraping the surface of the colonies with sterile plastic loops and sterile saline solution (0.85%), with subsequent filtration with filter paper Whatman No. 1 (Sigma-Aldrich, Missouri, USA) to separate hyphae and conidia. Suspensions were standardized with Neubauer chamber between 10^5^ and 10^6^ conidia per mL. Aliquotes of 850 μL were spread on the total surface of Petri dishes of 7 cm in diameter containing 8 mL of Sabouraud dextrose agar (SA) (Acumedia, São Paulo, Brazil). The fungi were cultivated at 30°C for 14 days.

Five replicates (fragments with 1 cm x 1 cm) of each culture on SA were cut with scalpel blade, deposited on filter paper (0.8 cm x 0.8 cm) Whatman No. 1 (Sigma-Aldrich, Missouri, USA) in Petri dishes and dehydrated in a drying oven at 50°C for 1 hour. Subsequently, dry agar portions were removed from the plates and submitted to FTIR-ATR analysis.

### FTIR-ATR analysis

Spectra of the five fragments of each fungal sample were acquired by attenuated total reflection (ATR) in a Spectrum 400 FT-IR/FT-NIR (Perkin Elmer) spectrometer. It was used an Universal ATR Sampling Acessory with top-plate with diamond/ZnSe crystal and one reflection (Perkin-Elmer, catalog number L1250050) whose ATR holder is horizontal. The acquisition range was 4000 to 650 cm^-1^ using a spectral resolution of 4 cm^-1^, force gauge 70 and 4 scans. Background was done reading the empty holder with clean crystal. The reduced number of scans was due to the high gain of the signal/noise ratio combined with the good interaction of the laser beam into the thick layer of the fungal culture biomass when under optimized force gauge.

### Chemometrics

The average spectra of each *F*. *pedrosoi* strain were obtained after correction and normalization of each replicate by amplitude (0–1, min-max normalization). The highest absorption band in each spectrum was considered 1 and the baseline 0. As a rule, all samples showed maximum absorption in the v-C-O band at 1045–1028 cm^-1^.

All chemometric analyses were made in the software Pirouette 4.0 (Infometrix). Figures were made in the software OriginPro70.

PLS was used to make a calibration model for MIC ITC and MIC ITC+TCZ determination. For this analysis, spectra from five replicates of each strain were used instead of the average spectrum to increase the prediction power of the model. Spectral variables (absorbance at each wave number) of each sample were pre-processed by calculating its 1^st^ derivative using the Savitzky-Golay algorithm (5 points) and the use of orthogonal signal correction (OSC) with one component. The dataset was divided into a calibration set (CS) and an external validation or prediction set (PS) using the systematic 1:1 alternating division, ordered from the lowest to the highest value of MIC, both for MIC ITC and MIC ITC+TCZ, resulting in two different CS/PS partitions for the two models. The minimum and maximum values were purposely included in CS. Thus, CS and PS were prepared with 18 strains each (total number of strains = 36). The maximum number of latent variables allowed in the PLS model was defined according to the recommendation of ASTM (American Society for Testing and Materials) E1655-05 using the following equation: N = 6 (A + 1), where N is the number of CS elements (N = 18) and A is the maximum number of latent variables allowed in the model, in order to avoid the occurrence of overfitting [14]. The figures of merit used to evaluate the CS were the coefficient of determination (R^2^) and the root mean square error of cross-validation (RMSECV) using leave-one out cross-validation (by steps of quintuplicates) as software guidelines. The PS values were determined by adding one strain (five replicates simultaneously) of *F*. *pedrosoi* at a time to the CS, identifying the predicted MIC values for each quintuplicate. PS performance was evaluated by R^2^ and root mean square error of prediction (RMSEP).

## Results and discussion

### FTIR-ATR spectra of *Fonsecaea pedrosoi*

**Fig 1:**
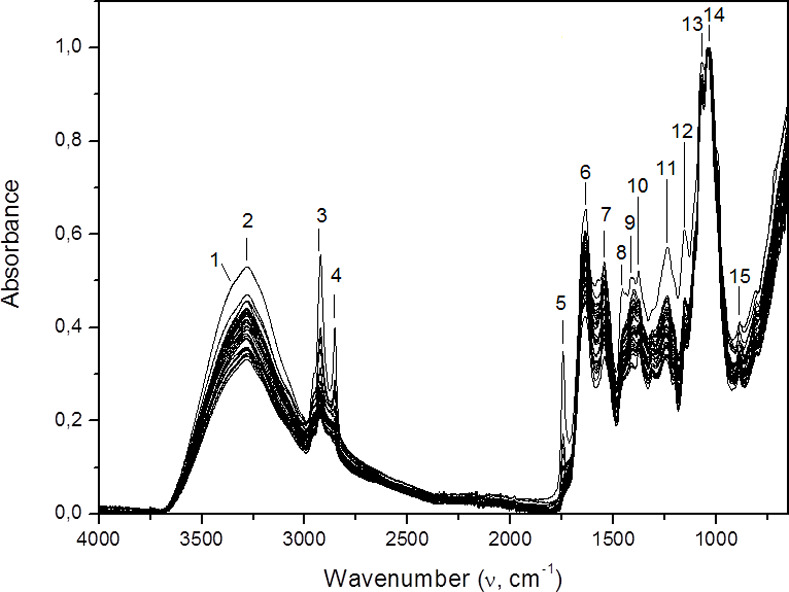
Total set of averaged FTIR-ATR spectra of *Fonsecaea pedrosoi* strains grown on Sabouraud medium. The numbers indicate the main bands found. Averaging from each strain was obtained calculating the arithmetic mean of absorption for each frequency of the five normalized min-max spectra.

The average spectra of the 36 strains of *F*. *pedrosoi* varied little and showed the typical pattern of filamentous fungi (Fig 1). The most prevalent bands (~1150, ~1070 and ~1030 cm^–1^, numbered 12, 13 and 14 in Fig 1) correspond to C˗O˗C, C˗O and C-C stretching dominated by ring vibrations of carbohydrates; they represent the glucans present in the fungus cell wall [15, 16]. Table 2 summarizes the characteristics and biomolecular attributions of the bands marked on Fig 1 [15, 17–19].

**Table 2:** Characteristics of the bands found in *F*. *pedrosoi* spectra.

Band number	Frequency (cm^-1^)	Vibrational mode/molecular bond	Biomolecular attribution
**1**	~3335	O-H and N-H stretching modes of amide A and amide B	**Proteins**
**2**	~3280		
**3**	~2920	CH_2_, CH_3_ asymmetric stretching	**Lipids**
**4**	~2850	CH_2_, CH_3_ symmetric stretching	
**5**	~1745	C = O stretching	
**6**	~1640	C = O of amide I (β-pleated sheet structures)	**Proteins**
**7**	~1550	N-H, C-N of amide II	
**8**	~1450	CH_2_, CH_3_ asymmetric and symmetric bending	**Lipids, proteins**
**9**	~1400	C = O symmetric stretching of COO^-^	**Lipids, amino acids**
**10**	~1380	Amide III	**Proteins**
**11**	~1240	P = O asymmetric stretching of phosphodiester	**Nucleic acids, phospholipids**
**12**	~1150	C-O-C, C-O, C-C stretching vibrations	**Carbohydrates**
**13**	~1070		
**14**	~1030		
**15**	~890	Aromatic ring vibrations	**Amino acids, nucleotides**

References: Naumann^15^, Salman *et al*.^17^, Salman *et al*.^18^, Lecellier *et al*.^19^

### PLS prediction model of MICs

**Fig 2:**
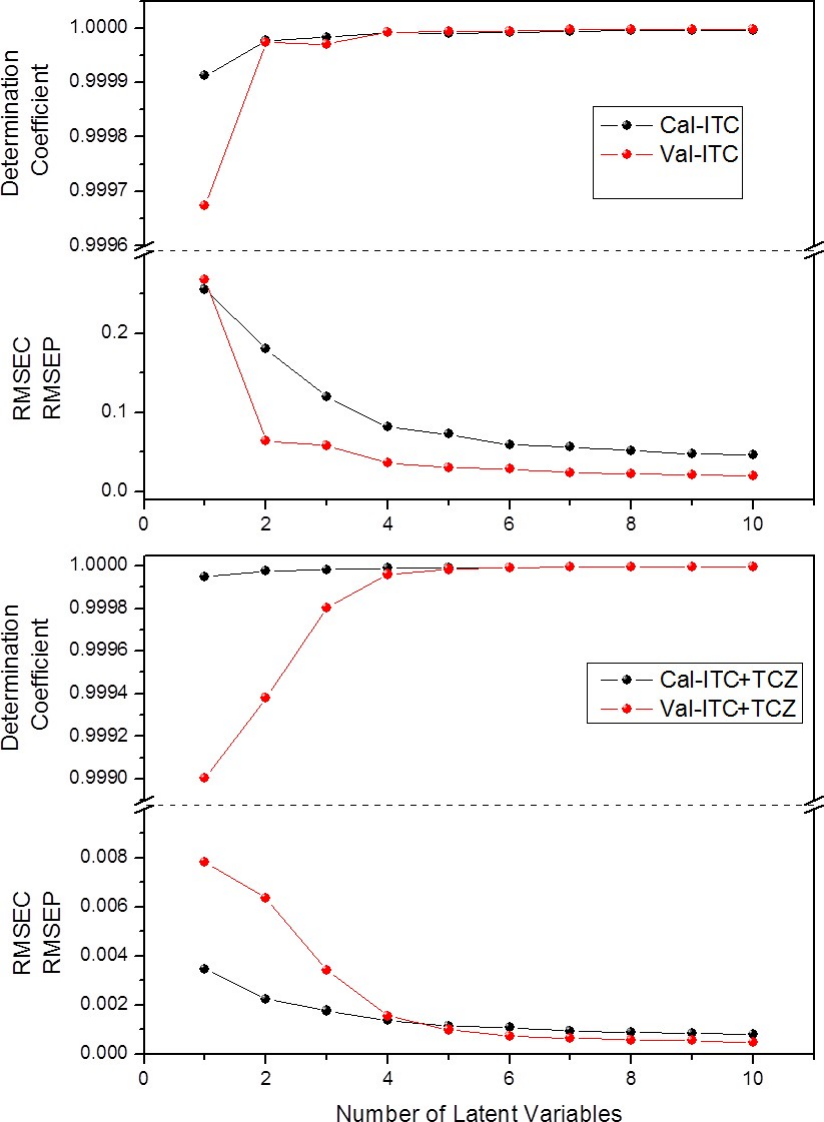
Figures of merit (RMSECV/RMSEP and respective coefficient of determination) profile of calibration and validation set of FTIR-ATR/PLS models of MIC ITC and MIC ITC+TCZ for *F*. *pedrosoi* samples investigated.

Different pre-processing protocols combined with PLS regression were evaluated. The best performance for predicting the MICs with ITC and ITC+TCZ was obtained with the application of normalization by amplitude combined with the 1st derivative and with one OSC component. The models obtained showed figures of merit (RMSECV/RMSEP and R^2^) compatible with high performance, even with one latent variable (Fig 2). However, the RMSEP error for ITC modeling was below the minimum MIC value quantified only with models with at least two latent variables. This is why two latent variables were considered for the construction and validation of PLS models (Table 3).

**Table 3:** Figures of merit of *F*. *pedrosoi* FTIR-ATR/PLS models of MIC ITC and MIC ITC+TCZ.

Parameter Set	LV	Range (mg/L)	ȳ±SD (mg/L)	R^2^	RMSECV RMSEP
MIC ITC					
CS	2	0.125–32	4.323 ± 9.938	0.999977	0.1803
			
PS	2			0.999973	0.0645
MIC ITC+TCZ					
CS	2	0.06–2	0.581 ± 0.354	0.999978	0.0022
			
PS	2			0.999382	0.0063

MIC = minimum inhibitory concentration; ITC = itraconazole; TCZ = tricyclazole; CS = calibration set. PS = prediction set; LV = number of latent variable; ȳ = mean; SD = standard deviation; R^2^ = determination coefficient; RMSECV = root mean square error of cross validation; RMSEP = root mean square error of prediction.

The reference values for MIC ITC and MIC ITC+TCZ were highly correlated with the values predicted from FTIR spectra, with R^2^ higher than 0.99 for both models. In the MIC ITC model, RMSEP was equal to 0.0645 mg/L, which is 51.6% of the lowest measured value; and in the MIC ITC+TCZ model, the RMSEP was equal to 0.0063 mg/L, i.e. 10.5% of the lowest measured value. Therefore, these values were lower than the lowest MIC measured in each category, which demonstrates the good performance of the model selected.

FTIR-ATR spectroscopy is effective in identification of microorganisms, as it provides highly specific fingerprints of a sample [20, 21]. The susceptibility or resistance of a microorganism to a given drug is defined by very subtle changes in the cells, and this technique combined with multivariate statistical analyses can detect these characteristics [11]. Only five studies have used FTIR-ATR to determine the sensitivity or resistance of microorganisms to certain drugs [8–11, 22]. However, although they obtained promising results, these studies evaluated only the use of FTIR-ATR with bacteria, for which there is already a high degree of standardization and a wide range of methods available for susceptibility testing [23]. Moreover, none of these studies used a PLS model.

This is the first study to propose the use of FTIR-ATR together with chemometrics to predict MICs for a species of filamentous fungus. The prediction was made directly from the fungus cultured in Sabouraud agar, without the need to expose the fungus to the antifungal tested. This indicates that the characteristics that determine susceptibility or resistance are intrinsic and are found in the structure of the cell wall and/or membrane [15]. Quintuplicates of each strain were read in different regions of the plate, since some strains did not grow homogeneously over the surface. These growth variations, which could pose a problem for reproducibility, as also non-specific between-strains variations, were corrected using one OSC component.

## Conclusions

The main advantages of the method proposed here are its ease of execution and lower cost compared to the reference methods. The limitations consist of the need for an initial set of MIC values (calibration set), which must be strictly defined following the standards of reference protocols to ensure a reliable prediction model. New samples with anomalous behavior should pass through the supervision method (M38) before they are included in the spectral calibration dataset for a new modeling update [24]. Finally, the information obtained here (using a Sabouraud agar culture) may not reflect the actual adaptations in RPMI 1640 medium or under *in-vivo* test conditions (where host defense mechanisms can generate other adaptation patterns in the presence of ITC). Studies of this nature are needed to enable a more complete interpretation of the clinical significance of these results. The present findings indicate that FTIR-ATR together with chemometrics allows the quantification of the degree of antifungal sensitivity of *F*. *pedrosoi* when challenged with itraconazole and/or tricyclazole. This technique can aid in the clinical management of infections caused by *F*. *pedrosoi* and is a promising alternative for studies of this nature with other filamentous fungi and other antifungal agents of clinical interest from the perspective of metabolomics.

## References

[1] Queiroz-Telles F, de Hoog S, Santos DWCL, Salgado CG, Vicente VA, Bonifaz A, et al Chromoblastomycosis. Clin Microbiol Rev. 2017; 30: 233–276. 10.1128/CMR.00032-16 27856522 PMC5217794

[2] Santos ALS, Palmeira VF, Rozental S, Kneipp LF, Nimrichter L, Alviano DS, et al Biology and pathogenesis of *Fonsecaea pedrosoi*, the major etiologic agent of chromoblastomycosis. FEMS Microbiol Rev. 2007; 31: 570–591. 10.1111/j.1574-6976.2007.00077.x 17645522

[3] Cunha MML, Frazen AJ, Seabra SH, Herbst MH, Vugman NV, Borba LP, et al Melanin in *Fonsecaea pedrosoi*: A trap for oxidative radicals. BMC Microbiol. 2010; 10: 80 10.1186/1471-2180-10-80 20233438 PMC2845570

[4] Bonifaz A, Paredes-Solis V, Saúl A. Treating chromoblastomycosis with systemic antifungals. Expert Opin Pharmacother. 2004; 5: 247–254. 10.1517/14656566.5.2.247 14996622

[5] Posteraro B, Sanguinetti M. The future of fungal susceptibility testing. Future Microbiol. 2014; 9: 947–967. 10.2217/fmb.14.55 25302953

[6] Balan V, Mihai C-T, Cojocaru F-D, Uritu C-M, Dodi G, Botezat D, et al Vibrational spectroscopy fingerprinting in medicine: from molecular to clinical practice. Materials. 2019; 12: 2884 10.3390/ma12182884 31489927 PMC6766044

[7] Bellisola G, Sorio C. Infrared spectroscopy and microscopy in cancer research and diagnosis. Am J Cancer Res. 2012; 2: 1–21. 22206042 PMC3236568

[8] Lechowicz Ł, Urbaniak M, Adamus-Białek W, Kaca W. The use of infrared spectroscopy and artificial neural networks for detection of uropathogenic *Escherichia col*i strains’ susceptibility to cephalothin. Acta Biochim Pol. 2013; 60: 713–718. 24432322

[9] Salman A, Sharaha U, Rodriguez-Diaz E, Shufan E, Riesenberg K, Bigio IJ, et al Detection of antibiotic resistant: Escherichia coli bacteria using infrared microscopy and advanced multivariate analysis. Analyst. 2017; 142: 2136–2144. 10.1039/c7an00192d 28518194

[10] Sharaha U, Rodriguez-Diaz E, Riesenberg K, Bigio IJ, Huleihel M, Salman A. Using infrared spectroscopy and multivariate analysis to detect antibiotics’ resistant *Escherichia coli* bacteria. Anal Chem. 2017; 89: 8782–8790. 10.1021/acs.analchem.7b01025 28731324

[11] Sharaha U, Rodriguez-Diaz E, Sagi O, Riesenberg K, Salman A, Bigio IJ, et al Fast and reliable determination of Escherichia coli susceptibility to antibiotics: Infrared microscopy in tandem with machine learning algorithms. J Biophotonics. 2019; 12: e201800478 10.1002/jbio.201800478 30916881

[12] Daboit TC, Magagnin CM, Heidrich D, Antochevis LC, Vigolo S, Meirelles LC, et al In vitro susceptibility of chromoblastomycosis agents to five antifungal drugs and to the combination of terbinafine and amphotericin B. Mycoses. 2014; 57: 116–120. 10.1111/myc.12111 23895037

[13] Shookohi GR, Badali H, Mirhendi H, Anasari S, Rezaei-Matehkolaei A, Ahmadi B, et al *In vitro* activities of luliconazole, lanoconazole, and efinaconazole compared with those of five antifungal drugs against melanized fungi and relatives. Antimicrob Agents Chemother. 2017; 61: e00635–17. 10.1128/AAC.00635-17 28848012 PMC5655049

[14] ASTM. Annual Book of ASTM Standards, Standards Practices for Infrared, Multivariate, Quantitative Analysis, ASTM International E1655-05. West Conshohocken, Pennsylvania; 2005.

[15] Naumann D. Infrared Spectroscopy in Microbiology In: Meyers RA, editor. Encyclopedia of Analytical Chemistry. Chichester: John Wiley & Sons Ltd; 2000 pp. 102–131.

[16] Nie M, Luo J, Xiao M, Chen J, Bao K, Zhang W, et al Structural differences between *Fusarium* strains investigated by FT-IR spectroscopy. Biochemistry (Moscow). 2007; 72: 61–67. 10.1134/s0006297907010075 17309438

[17] Salman A, Tsror L, Pomerantz A, Moreh R, Mordechai S, Huleihel M. FTIR spectroscopy for detection and identification of fungal phytopathogenes. Spectroscopy. 2010; 24: 261–267.

[18] Salman A, Lapidot I, Pomerantz A, Tsror L, Shufan E, Moreh R, et al Identification of fungal phytopathogens using Fourier transform infrared-attenuated total reflection spectroscopy and advanced statistical methods. J Biomed Opt. 2012; 17: 017002 10.1117/1.JBO.17.1.017002 22352668

[19] Lecellier A, Mounier J, Gaydou V, Castrec L, Barbier G, Ablain W, et al Differentiation and identification of filamentous fungi by high-throughput FTIR spectroscopic analysis of mycelia. Int J Food Microbiol. 2014; 168–169: 32–41. 10.1016/j.ijfoodmicro.2013.10.011 24231128

[20] Shapaval V, Møretrø T, Suso H-P, Asli AW, Schmitt J, Lillehaug D, et al A high-throughput microcultivation protocol for FTIR spectroscopic characterization and identification of fungi. J. Biophotonics. 2010; 3: 512–521. 10.1002/jbio.201000014 20414905

[21] Wenning M, Scherer S. Identification of microorganisms by FTIR spectroscopy: Perspectives and limitations of the method. Appl Microbiol Biotechnol. 2013; 97: 7111–7120. 10.1007/s00253-013-5087-3 23860713

[22] Sockalingum GD, Bouhedja W, Pina P, Allouch P, Mandray C, Labia R, et al ATR-FTIR spectroscopic investigation of imipenem-susceptible and -resistant *Pseudomonas aeruginosa* isogenic strains. Biochem Biophys Res Commun. 1997; 232: 240–246. 10.1006/bbrc.1997.6263 9125140

[23] Khan ZA, Siddiqui MF, Park S. Current and emerging methods of antibiotic susceptibility testing. Diagnostics. 2019; 9: 49 10.3390/diagnostics9020049 31058811 PMC6627445

[24] CLSI. M38 –Reference method for broth dilution antifungal susceptibility testing of filamentous fungi. 3rd ed. Wayne, Pennsylvania: Clinical and Laboratory Standards Institute; 2017.

